# In-office magnetic resonance imaging (MRI) equipment ownership and MRI volume among medicare patients in orthopedic practices

**DOI:** 10.1186/s13561-015-0068-0

**Published:** 2015-10-20

**Authors:** Robert L. Ohsfeldt, Pengxiang Li, John E. Schneider

**Affiliations:** 1 School of Public Health, Texas A&M University, MS 1266, College Station, TX 77843-1266 USA; 2 General Internal Medicine, Perelman School of Medicine, University of Pennsylvania, Philadelphia, PA 19104-6218 USA; 3 CEO, Avalon Health Economics, 20 South Street, Suite 2B, Morristown, NJ 07960 USA

**Keywords:** Medicare, Physician self-referral, Orthopedic practice, Transactions costs

## Abstract

**Background:**

Concerns have been raised about physician ownership of onsite advanced imaging equipment as allowed under Stark laws by the in-office ancillary service exception (IOASE).

**Methods:**

A web-based survey of orthopedic practices in the United States was used to assign a first date of onsite MRI capacity acquisition (if any) to specific orthopedic practices. Medicare claims data for 2006–2010 was obtained for providers in orthopedic practices acquiring onsite MRI capacity and in matched orthopedic practices without an onsite MRI over the same period of time. Multivariate regression was used to estimate the change in provider Medicare MRI volume one year before and one year after the onsite MRI acquisition year for providers in MRI practices compared to providers in propensity-score matched non-MRI practices.

**Results:**

In all of the MRI volume change models estimated, the association between onsite MRI acquisition and the change in provider Medicare MRI volume (one-year post-onsite-MRI-acquisition less one year pre-acquisition) was consistently small and not statistically significant. This lack of association was robust to changes in model specification in terms of types of MRI exams considered, specific covariates included in the multivariate model, or the process used to confirm individual provider affiliation with study practices in study years.

**Conclusions:**

Our analysis of Medicare claims data provides no empirical support for the proposition that acquisition of onsite MRI capacity within an orthopedic surgery practice induces an increase in the rate of MRI use for Medicare patients among practice providers, relative to physicians in practices without MRI capacity over the same time period.

## Background

Considerable concern has been expressed about the effects of physician ownership of imaging equipment on the use of such services in the United States [1–4]. A series of laws known as “Stark Laws” (named for the law’s primary sponsor, United States Congressman Pete Stark) generally prohibit physicians from referring patients covered by Medicare (a universal public insurance program for persons age 65 or older) for certain “designated health services” if the referring physician or his/her family has a financial relationship with the service provider. The first of these laws (“Stark I”), effective in 1992, banned referral of Medicare patients to provider-owned clinical laboratories. Effective in 1998, “Stark II” expanded the self-referral ban to a number of additional ancillary health services, and extended the self-referral ban to patients covered by Medicaid (a public insurance program for low income individuals). Finally, effective in 2007, “Stark III” provided additional regulatory guidance for compliance, such as defining specific provider compensation arrangements as analogous to ownership interests [5, 6].

The Stark Law restrictions on physician self-referral were intended to avoid the financial incentives for physicians to increase the volume of referrals for ancillary services, particularly with physician ownership of imaging service capacity [7–17]. However, many factors affect decisions about which patients receive imaging services. Carey and Garrett found that the use of CT and MRI exams for low back pain patients was associated with patient characteristics, such as baseline functional status [18]. In a randomized controlled trial of patients with low back pain, Gilbert and colleagues found those who received “early” imaging had better outcomes than those receiving delayed imaging [19]. If onsite imaging advances the timing of imaging or otherwise enhances the appropriate use of imaging in treatment, onsite imaging may improve the quality of care. Some have questioned whether the lower rate of referral among physicians without ready access to imagining capacity represents underuse rather than overuse by physicians with such capacity [20]. Indeed, the rationale for the “In-Office Ancillary Services Exception” (IOASE) to the Stark restriction relates to the potential benefits of onsite ancillary service availability [5].

The incentives for physician practices to acquire onsite imaging capacity extend beyond the indirect payment from a referral to a physician-owned service in a fee-for-service (FFS) payment system. The relationship between a physician practice and ancillary services represents a “vertical relationship,” which can be organized through market-based contractual arrangements or through vertical integration [21, 22], i.e., direct practice ownership as permitted by IOASE. Orthopedic practices without an onsite MRI typically refer patients to a shared MRI facility, often offered through a hospital outpatient department (HOPD) proximate to the practice location, but practices relying on shared facilities have less control over the scheduling of MRI exams.

Thus, the choice of using onsite or shared MRI equipment is a variant of the classic “make or buy” decision in organizational economics, which is mainly influenced by scope economies and transaction economies [23–29]. The make-or-buy decision has been studied extensively in the context of transaction cost economics, which posits that the boundaries of organizations are in large part a function of the nature of the business transacted, where relatively complex transactions are more efficiently organized in settings that feature stronger administrative controls, such as ownership [23, 27, 29]. In the market for medical care, consumer transaction costs are the costs incurred to the consumer to complete a transaction, including the time necessary to implement informed choice, such as evaluating, choosing and locating a care provider, as well as the time spent directly obtaining the services [22]. Consumer transaction costs are expected to be lower in the case of onsite MRI availability because patients may be able to economize on identifying, vetting, locating and traveling to a provider [30]. In addition, there are several potential convenience-related benefits associated with onsite availability, including easier scheduling, enhanced adherence to treatment plans [31, 32], and “one-stop shopping” [33–35]. Likewise, monitoring costs may be reduced via onsite MRI capacity, to the extent it permits practices to improve supervision of the quality of care, and allows for better coordination among patients, physicians, and ancillary services, and to provide incentives for patients to adhere to recommended treatment plans [36].

Opponents of IOASE contend that the purported benefits of onsite availability are non-existent or overstated [37], instead focusing on the role of asymmetrical and imperfect information, which may allow providers to “induce” demand for ancillary services [8–17]. The potential impact on the extent of demand inducement resulting from physician ownership of imaging services under FFS payment relates to the magnitude of the indirect payment to providers from imaging service ownership, which would be analogous to an equivalent increase in the direct provider payment for professional services [22]. The impact of this incentive is muted by payer policy which often requires pre-authorization or pre-certifications, thus limiting provider discretion over the provision of imaging services [38, 39].

Those advocating an end to IOASE point to a number of studies concluding the financial incentives from physician self-referral causes an increase in the volume of services provided under FFS payment so large as to outweigh any benefits [8–17], though some suggest that movement away from FFS payment would be a superior solution compared to ending IOASE [40]. However, most of these studies do not provide adequate adjustment for incentives beyond self-referral for practices to acquire onsite services, which is a likely source of bias toward finding a positive association between MRI acquisition and MRI volume.

The present study addresses this methodological limitation in the existing literature by using Medicare claims data to assess the extent of differences in MRI exams for Medicare patients among providers in orthopedic practices before and after their practice acquired onsite MRI capacity, compared to physicians in matched orthopedic practices without onsite MRI over the same period of time.

## Methods

A persistent challenge in the literature on this subject is the limited data on the extent and timing of physician practice acquisition of imaging capacity. The present study used a web-based survey of orthopedic practices in the United States to determine the date of onsite MRI capacity acquisition, or the absence of onsite MRI capacity, to facilitate a comparison of MRI use with/without onsite MRI capacity. A practice-level propensity score (PS) matching approach was used to match orthopedic practices with onsite MRI to non-MRI practices. Multivariate regression models were used to examine the change in Medicare MRIs per Medicare patient one year before and one year after the onsite MRI acquisition year for providers in MRI practices compared to providers in non-MRI comparison practices.

### Practice survey data

A survey of orthopedic practices in the United States was initiated in July 2012 with the support of the American Academy of Orthopaedic Surgeons (AAOS) to determine the date of first acquisition onsite MRI equipment (if any), and general information about the practice. Details about the administration of the AAOS practice survey have been reported elsewhere [41].

For practices reporting onsite MRI capacity, respondents were asked to report the number of practice providers (and their UPIN/NPI numbers) authorized to order an MRI as of the year of their first onsite MRI acquisition. All non-MRI practice respondents reported the number of current providers in the practice authorized to order MRI exams (and their UPIN/NPI numbers).

By September 2012, the orthopedic practice survey was closed with a total of 770 responses received. Eliminating duplicate and incomplete responses yielded 740 practice responses. An additional 185 practices did not report provider ID numbers (167 [90 %] of these reported no onsite MRI capacity) and thus were excluded from the practice sample used for PS matching of onsite MRI and comparison non-MRI practices.

### Selection of MRI and non-MRI practices

At the time of this study, the most recent full year of Medicare claims data available was for 2010. To assure a full year of Medicare claims data before and after MRI acquisition, 63 practices which reported a first MRI acquisition in 2007, 2008, or 2009 were classified as MRI “case” practices. Similarly, 465 practices without an onsite MRI by December 31, 2010, or practices which acquired an onsite MRI after January 1, 2011, were classified as non-MRI practices.

Preliminary confirmation of the respondent-reported physician ID numbers was obtained by using the survey-reported physician ID numbers to the NPI/UPIN crosswalk file to get a UPIN number (for physicians with an NPI in the survey) or NPI number (for physicians with a UPIN in the survey). Next, we merged the survey physician UPIN/NPI numbers with the CMS National Plan and Provider Enumeration System (NPPES) Full Replacement Monthly NPI File [42] for the MRI acquisition year (for MRI practices) or 2012 (for non-MRI practices).

Comparing the city and state of the provider’s business mailing address from NPPES to the survey reported city and state of practice address revealed the states did not match for more than 50 % of the physician ID numbers for 195 of the survey practices. These 14 MRI practices and 181 non-MRI practices were excluded from the practices considered for inclusion in the final sample of practices (see Table 1). In addition, we excluded 172 practices not serving Medicare beneficiaries and all providers without valid UPIN/NPI.

**Table 1:** Survey practices and sample physicians

*A: Number of survey practices and physicians by cohort*			
*MRI acquisition year*	*Number of practices*	*Practices with >50 % physician ID match*	*Number of physicians*
Comparison			
Without MRI thru 2010	442	265	1790
2011-2013	23	19	220
Total	465	284	2010
Treatment			
2007	30	21	276
2008	15	12	206
2009	18	16	283
Total	63	49	765

*Sources: AAOS Survey Data, 2012; CMS NPPES Downloadable File* [39]*; see text*

For the resulting sample of 32 MRI practices and 129 non-MRI practices, we used a propensity score (PS) approach to identify specific non-MRI (comparison) practices to be matched to specific MRI (case) practices. The PS matching approach was originally developed in part to enhance the efficiency of sampling comparison observations to be included in the study sample over random sampling from a large pool of potential observations [43]. The first step in the PS matching approach is to estimate a model to predict the likelihood of onsite MRI acquisition for individual practices based on various practice characteristics – specifically practice characteristics that might also affect the volume of MRI exams performed by practice providers.

We used a logistic regression model predicting the likelihood of onsite MRI acquisition which included as predictor variables the number of providers in the practice, practice payer mix (Medicare revenue share), number of Medicare beneficiaries they served, number of providers with valid UPIN or NPI), percentage of providers in the same city during our study years, and dummy variables for Census region (model results not reported). The Hosmer–Lemeshow *χ* ^2^ test statistic for the model is 21.8 (*p* < 0.01), with a c-statistic of 0.827 and McFadden’s R-squared of 0.30. The common support for the PS model (in terms of predicted probabilities) covers the range of 0.056 to 0.921, with 76 % of practices (123 out of 161) in this range. Only the practice size variables (number of providers in the practice, number of Medicare beneficiaries served, and number of providers with valid UPIN or NPI) were statistically significant in the model (*p* < 0.01)

The logistic regression index value (i.e., **Xβ**) for each practice was used as a practice-level propensity score for onsite MRI acquisition. For PS matching of MRI to non-MRI practices, there is a classic trade-off between the degree to which PS matching achieves “balance” across covariates for case and comparison practices and the number of case practices retained in the PS-matched sample to be used in the analysis [44]. In this case, because MRI practices are fundamentally different from non-MRI practices in terms of a key practice characteristic (specifically, practice size), restricting the matching of non-MRI practices to comparable MRI practices based on an exact or near exact PS would have resulted in a very small sample of matched case-comparison practices. Adding more covariates to the PS model would not enhance the prospects for more precise PS matches given the predominance of practice size in predicting MRI acquisition.

To address the trade-off between covariate balance and sample size, we used PS caliper matching to avoid selecting a non-MRI practice as a match for an MRI practice when the practices were too dissimilar to constitute a reasonable match, while retaining a reasonable sample size. Specifically, we used one-to-one PS caliper matching (without replacement), with the caliper restricting the acceptable difference in PS to be less than 25 % of the standard deviation of the PS distribution across all practices [45]. By imposing this PS caliper restriction, 23 MRI practices and 23 matched non-MRI comparison practices were identified, with a total of 252 and 181 affiliated providers, respectively (Table 2).

**Table 2:** Survey practices and sample physicians

*B: Number of physicians and practices among treatment and control group*						
	Number of practices			Number of physicians		
	Comparison	Treatment	Total	Comparison	Treatment	Total
2007	11	11	22	67	100	167
2008	4	4	8	17	35	52
2009	8	8	16	97	117	214
Total	23	23	46	181	252	433

*Sources: AAOS Survey Data, 2012; CMS NPPES Downloadable File* [39]*; see text*

### Medicare claims data

**Fig. 1:**
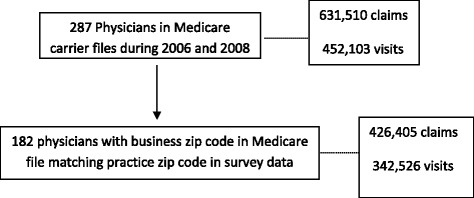
Flow Chart of Sample Selection for 2007 Cohort

Three years of Medicare Part B utilization data were obtained for each of the 433 physicians from the three MRI “treatment” cohorts (2007, 2008, and 2009) and the three matched non-MRI comparison cohorts. For example, for each of the 100 physicians in the 2007 MRI treatment group and each of 67 physicians in the 2007 non-MRI comparison group, we accumulated all Medicare claims containing each individual UPIN/NPI for one year before and one year after the MRI acquisition cohort year. Specifically, we obtained all patient claims from Medicare carrier files for 2006, and 2008 associated with 167 physician UPIN/NPIs. With duplicate UPIN/NPIs associated with physicians with multiple practice locations, there were a total of 287 physician IDs (UPIN/NPIs) in the “finder file” (used to link providers to their claims) for calendar years 2006 and 2008 (i.e., one year before and one year after 2007), with 631,510 claims and 452,103 Medicare patient visits in the Medicare carrier file with one of the 287 UPIN/NPIs. Among these 287 UPIN/NPIs, 182 UPIN/NPIs had a business zip code in Medicare carrier file that matched the practice zip code in the AAOS survey (see Fig. 1). The sample of physicians with UPIN/NPI zip codes that match the AAOS survey zip code are used as the principal sample for the analysis of patterns of MRI use in the Medicare claims data.

An analogous approach was used to aggregate Medicare claims data for the physicians in the 2008 and 2009 cohorts. Specifically, the pre-MRI year Medicare claims data are for the calendar year 2007 and 2009 for the 2008 and 2009 cohorts, respectively, and the post-MRI year Medicare claims data are for the calendar year 2009 and 2010 respectively.

Despite our efforts to use all available CMS data to confirm the practice affiliation of providers obtained from the AAOS practice survey data, the possibility of errors in the assignment of specific providers to specific practices at the time of first onsite MRI acquisition remains. To assess the extent of any assignment errors, all 46 practices included in the final sample of matched onsite MRI and non-MRI practices were re-surveyed. The MRI practices were asked to confirm that the practice acquired its first onsite MRI in the indicated MRI year (e.g., 2008 for MRI practices in the 2008 cohort), and non-MRI practices were asked to confirm that the practice did not have onsite MRI capacity in any of the study years for that practice (e.g., 2007–2009 for a non-MRI practice in the 2008 cohort). The re-survey instrument also provided a list of UPIN/NPI numbers specific to each of the 46 practices (obtained from the initial survey). Practices were asked to confirm whether the listed providers were affiliated with the practice during all of the specific study years for that practice (e.g., 2007–2009 for a practice with MRI year 2008, or a non-MRI practice matched to a 2008 MRI practice).

A total of 20 of the 46 practices responded to the re-survey (46 % response rate). All of the responding practices confirmed that the MRI or non-MRI status in the survey was correct. The respondents also confirmed that about 90 % of the provider ID numbers from the original survey were affiliated with the practice in both the pre- and post-MRI year for the practice. While the results of the re-survey suggest that provider timing assignment errors in the principal provider sample were not common, all regression models using the principal provider sample were re-estimated using a restricted sample of providers with a confirmed practice affiliation for the pre- and post-MRI years.

### Analytic approach

The unit of analysis for the Medicare claims data analysis is the individual physicians affiliated with the MRI treatment practices and the matched non-MRI comparison practices. The analysis focuses on the difference in the volume of MRI exams ordered by each physician during the calendar year after the year of onsite MRI acquisition and the volume of MRI exams ordered by the same physician during the calendar year before MRI acquisition. The intent is to assess the “steady state” volume of MRI exams with and without onsite MRI, as the volume of MRI exams immediately after the acquisition of onsite MRI capacity may be atypical if practices work off “pent up” demand for MRI exams when the onsite MRI capacity first becomes available.

The analytic approach makes use of a multivariate regression model of the general form

1 $$ \Delta {\mathrm{MRI}}_{i,j,r} = \left({\mathrm{MRI}}_{i,j,r,t+ 1} - {\mathrm{MRI}}_{i,j,r,t- 1}\right) = \upalpha + \upbeta\ {\mathrm{Onsite}}_{j,r,t} + \upphi\ {\mathrm{Practice}}_{j,r,t} + \uppsi\ {\mathrm{Area}}_{r,t} + {\upepsilon}_{i,j,r} $$

In Eq. (1), the term “∆MRI_*i*,*j*,*r*_” indicates the difference in the volume of MRI exams in the Medicare claims data for an individual physician (*i*) in a specific practice (*j*) located in a specific county (*r*) for one year post-onsite-MRI acquisition (*t + 1*) and one year pre-onsite-MRI acquisition (*t-1*). For physicians in the matched non-MRI comparison practices, the MRI acquisition year for the matched MRI practice (*t*) is used as a pseudo-MRI year to define the pre- and post-MRI-year volume of MRI exams. The term “Onsite_*j*,*r*,*t*_” is a binary variable equal to one for physicians in practices acquiring onsite MRI capacity in year *t* and zero for physicians in the matched non-MRI practices.

The modeling approach is a variant of the familiar “differences in differences” approach [46]. By focusing on the change in the volume of MRI exams for individual physicians, each physician acts as his or her own “control,” in that any specific characteristics of the individual physician (e.g., practice style, patient case mix) that might influence the physician’s use of MRI exams but remain essentially constant over the 3 year pre/post period will “difference out” when examining the change in the volume (post-pre) onsite MRI acquisition. Thus, the dependent variable is only affected by factors that vary over time. Beyond the change in onsite MRI status, general market conditions for orthopedic services could have changed over the pre- and post-MRI periods. Thus, a multivariate model is estimated that also adjusts for differences between MRI and non-MRI practices in practice characteristics (“Practice_*j,r*,*t*_”) and county-level practice area characteristics (“Area_*r*,*t*_”) that remain after PS matching. Finally, α, β, ϕ, and ψ in Eq. (1) represent parameters to be estimated by the regression model, and ϵ represents an error term. The estimation procedure used accounts for the likely correlation in errors among physicians in the same practices.

As noted, a PS matching procedure was used to provide a rationale for the selection of the MRI and comparison non-MRI practices to be used to collect Medicare claims data for the providers in the selected MRI and non-MRI practices. If PS matching had achieved an exact or near exact match between case and comparison practices, differences in observed practice characteristics between the physicians in the treatment and comparison groups might have been negligible, making covariate adjustment for practice characteristics in a multivariate regression unnecessary. However, PS matching of MRI practices to non-MRI practices is approximate in this application. Coupled with the fact that the level of analysis is the individual providers in the matched practices, some significant differences between the practice characteristics of the physicians in the MRI practices and physicians in matched non-MRI practices remain, as shown in Table 3. Physicians in MRI-acquiring practices had higher Medicare MRI volume than physicians in non-MRI practices both one year before and one year after the MRI acquisition year. The MRI-acquiring practices were larger (in terms of number of providers) and were located in areas experiencing growth in per capita income, compared to non-MRI practices. Given these differences, some covariate adjustment in a multivariate regression model may be needed [47]. Thus, we estimate alternative specifications of Eq. (1) with and without different categories of covariates included in the model.

**Table 3:** Sample means for physician practice sample, by on-site MRI status

	All	MRI	No MRI
	[*n* = 433]	[*n* = 252]	[*n* = 181]
*MRI Volume* ^*a*^ *(% Medicare Visits)*			
Pre-MRI year	1.151	1.460	0.595
Post-MRI year	1.312	1.530	0.919
∆(Post-Pre)	0.161	0.071	0.324
*Ortho-MRI Volume* ^*b*^ *(% Medicare Visits)*			
Pre-MRI year	1.066	1.391	0.482
Post-MRI year	1.239	1.478	0.809
∆(Post-Pre)	0.173	0.087	0.327
*MRI Year (%)*			
2006	38.55	44.72	27.43
2007	26.54	31.21	18.14
2008	34.91	24.08	54.42
*Number of providers (%)*			
1-2	2.37	1.47	3.98
3-5	6.64	5.90	7.96
6-10	26.86	12.53	52.65
>10	64.14	80.10	35.40
*Practice Payer Mix (%)*			
Medicare	27.07	26.13	28.77
Private Insurance	46.90	47.83	45.22
Workers’ Comp	12.30	12.10	12.64
Other	13.74	13.94	13.38
*Area Characteristics (Post-Pre)*			
∆Per capita income ($1000s)	1.211	1.916	−0.058
∆Pop age 65+ (%)	0.449	0.467	0.417
∆Unemployment (%)	2.860	2.461	3.578
∆MDs/1000 Population	−0.0018	−0.0045	0.0032

*Sources: Medicare Claims Data; AAOS Survey Data, 2012; Area Resource File (see text)*

^a^HCPCS codes 71552, 73218, 73718, 74183, 77059, 70543, 70551, 70553, 72141, 72146, 72148, 72156, 72157, 72158, 72195, 72197, 73220, 73221, 73222, 73223, 73720, 73721, 73722, 73723, 70336, 70540, 70542, 70552, 71550, 71551, 72142, 72147, 72149, 72196, 73219, 73719, 74181, 74182, or 77084

^b^HCPCS codes 72141, 72146, 72148, 72156, 72157, 72158, 72195, 72197, 73220, 73221, 73222, 73223, 73720, 73721, 73722, or 73723

The primary measure of “∆MRI_*i*,*j*,*r*_” is the difference in the total number of Medicare MRI exams (post-pre) ordered by each physician as a percentage of all Medicare outpatient visits for each physician. (See Table 3 for specific HCPCS codes defining MRI exams.). An alternative measure focuses on the post/pre difference in MRI exams with diagnosis codes indicative of orthopedic conditions (“Ortho-MRI”) as a percentage of all Medicare outpatient visits for each physician (see Table 3). We also analyze the post-pre difference in the absolute (total) number of Medicare MRI exams and Medicare orthopedic-MRI exams for each physician.

All multivariate regression models were estimated using Stata Version 13 (http://www.stata.com/stata13/), employing the “cluster” option (to account for physicians in the sample affiliated with the same practice) and the “robust” standard error option (to account for other potential departures from homoscedasticity by using the Huber-White robust standard error estimator).

## Results

Table 4 provides model estimates of the effect of onsite MRI acquisition (“Onsite MRI”) on the change in total Medicare MRI exams as a percentage of total Medicare outpatient visits for specific physicians over the post/pre MRI year period. Column 1 of Table 4 reports the estimated impact onsite MRI capacity acquisition on the change in Medicare MRI volume as a percentage of Medicare visits in a regression model with no covariate adjustment (other than MRI cohort year). The model specification in column 2 adds measures of practice size as covariate adjusters, column 3 also includes practice payer mix variables, and column 4 adds the post-pre change in levels of county-level practice area characteristics.

**Table 4:** Estimated difference in percent medicare visits for MRI exams for physicians Post/Pre Onsite MRI acquisition relative to physicians without onsite MRI, 2007–2009 cohorts

∆MRIs as % Visits	Model 1		Model 2		Model 3		Model 4	
	Coeffi-cient	*p*-value	Coeffi-cient	*p*-value	Coeffi-cient	*p*-value	Coeffi-cient	*p*-value
*Onsite MRI*	−0.330	0.486	−0.139	0.753	−0.237	0.507	−0.468	0.161
*MRI Year*								
2007	Reference	--	Reference	--	Reference	--	Reference	--
2008	1.569	0.063	1.570	0.068	0.817	0.125	2.175	0.038
2009	0.424	0.454	0.567	0.323	0.227	0.533	1.157	0.067
*Number of providers*								
1-2	--	--	Reference	--	Reference	--	Reference	--
3-5	--	--	−1.930	0.088	−1.121	0.398	−1.086	0.448
6-10	--	--	−2.199	0.037	−2.142	0.076	−1.892	0.095
>10	--	--	−2.395	0.023	−2.015	0.072	−1.907	0.083
*Practice Payer Mix (%)*								
Medicare	--	--	--	--	0.0734	0.006	0.0926	0.010
Private Insurance	--	--	--	--	0.0660	0.004	0.0644	0.006
Workers’ Comp	--	--	--	--	0.0289	0.511	0.0442	0.353
Other	--	--	--	--	Reference	--	Reference	--
*Area Characteristics*								
∆Per cap inc ($1000s)	--	--	--	--	--	--	0.072	0.278
∆Pop age 65+ (%)	--	--	--	--	--	--	0.516	0.198
∆Unemployment (%)	--	--	--	--	--	--	−0.246	0.216
∆MDs/1000 Pop	--	--	--	--	--	--	−1.215	0.418
*F-Statistic (p-value)*	1.37	0.266	1.81	0.125	2.80	0.013	2.93	0.007

*Sources: see text*

The point estimate for the coefficient of the onsite MRI variable in each of these alternative regression model specifications is negative, which suggests the change in Medicare MRI volume for providers in MRI practices was lower than the change for non-MRI practices over the same time period, but all of the estimated coefficients are small in magnitude and not statistically significant (*p* > 0.05).

Focusing briefly on estimated coefficient values for other covariates included in the model reported in column 4, the estimated coefficients of the MRI cohort year variables suggest that the change in MRI volume for providers in the 2008 cohort was 2.2 percentage points greater than the change for providers in the reference-category 2007 cohort (*p* = 0.038), adjusting for other variables included in the model. A 1 percentage point greater Medicare share in the practice payer mix was associated with a 0.09 percentage point greater change in provider Medicare MRI volume (*p* = 0.010), and a 1 percentage point greater private insurance share in the practice payer mix was associated with a 0.06 percentage point greater change in provider Medicare MRI volume (*p* = 0.006). None of the remaining estimated coefficients were statistically significant at the *p* < 0.05 level.

To assess whether the finding of a lack of association between onsite MRI acquisition and changes in the volume of Medicare MRI exams is robust to model specification changes, models were estimated using four alternative measures of the change in provider MRI exam volume: 1) the change in MRI exams as a percentage of all Medicare patient visits; 2) the change in orthopedic-related MRI exams as a percentage of all Medicare patient visits; 3) the absolute change in the number of MRI exams; and 4) the absolute change in the number of orthopedic-related MRI exams.

We also estimated models using the principal study sample and an alternative subsample of providers confirmed by the AAOS practice re-survey to have been practicing in the study practices during both study years. Point estimates of the coefficient of the onsite MRI variable and their associated *p*-values (for a two-tailed test of the null hypothesis that the true coefficient equals zero) across these alternative model specifications are summarized in Table 5. [Full model results are available on request].

**Table 5:** Summary of estimated coefficient for “Onsite MRI” for alternative measures of the difference in medicare MRI volume and alternative provider samples

*MRI volume change measure*	*Principal provider sample (N = 433)*		*Confirmed provider sample (N = 371)*	
	“Onsite MRI”	*p*-value	“Onsite MRI”	*p*-value
∆MRIs as % Visits	−0.468	0.161	−0.604	0.273
∆Ortho MRIs as % Visits	−0.399	0.210	−0.787	0.128
∆Number of MRIs	2.165	0.420	1.591	0.310
∆Number of Ortho MRIs	0.102	0.977	0.152	0.507

*Sources: see tex*

None of the point estimates of the onsite MRI coefficient are statistically significant (*p* > 0.1) across all of alternative model specifications reported in Table 5. Results using the principal provider sample are similar (in terms of coefficient point estimates) to results using a sample restricted to providers with their practice location during the study years confirmed by the practice re-survey. Thus, any potential errors in the assignment of specific physicians to specific practices appear to be too infrequent to have a substantive impact on model results.

## Discussion

Economic theory predicts, and our results confirm, practices using imaging more intensively were more likely to acquire onsite MRI capacity (i.e., acquiring practices had higher MRI volume than non-MRI practices before MRI acquisition). This creates a sample selection (or endogeneity) issue when attempting to assess the causal impact of onsite MRI acquisition on MRI volume. By using a differences-in-differences model focusing on the change in MRI volume for individual physicians, any individual physician or practice characteristics (observed or unobserved) potentially affecting MRI volume that are invariant over the pre- and post- time periods “difference out” when analyzing the change in MRI volume. Covariate adjustment using proxy measures of physician “practice style” is not needed. Our model also adjusts for changes in observable practice area characteristics over time. To the extent unobservable time-varying factors exist, such factors are likely to affect the demand for imaging services and the likelihood of onsite MRI acquisition in the same direction. Thus, any remaining bias in our analysis relating to the sample selection issue would be toward finding a positive association between MRI acquisition and MRI volume.

None of our model results suggest any substantive change in Medicare MRI volume one-year post- onsite-MRI-acquisition and one-year pre-onsite-MRI-acquisition for physicians in MRI-acquiring practices relative to physicians in the non-MRI comparison practices. This finding is inconsistent with results reported in much of the literature focused on the issue of “self-referral” for imaging services.

The differences in findings may relate to differences in research designs, particularly as they relate to sample selection issue, and the specific measures of MRI acquisition used across studies. Some existing studies rely on proxy measures of the existence or size of ownership interests in specific ancillary services for individual physicians due to a lack data for specific provider interests. For example, close to a dozen published studies (e.g., Hughes et al. [12], Mitchell [13]) use an individual physician’s referral patterns to “impute” physician ownership status for individual physicians. Specifically, physicians with a relatively high share of their overall referrals going to a physician-owned facility are simply assumed to have ownership interest in the facility. These studies provide little or no evaluation of the validity of this imputation process for identifying individual physician ownership status, but even if approximately valid, the use of an imputed ownership status indicator based on patterns of referrals to predict patterns of referrals presents what should be a rather obvious and substantial threat to the validity of any resulting inferences about the causal effect of ownership status on referral volume. In contrast, our analysis uses direct and verified measures of access to onsite MRI capacity for individual providers.

A simple cross-sectional design is used in close to a dozen published studies, including Hillman et al. [10] and Paxton et al. [16]. These studies compare imaging volume for physicians with and without ownership interest in imaging capacity, not before and after the acquisition of ownership interest. Our results indicate that the physicians in practices acquiring onsite MRI capacity had higher MRI volume before MRI acquisition than physicians in similar practices that did not subsequently acquire onsite MRI capacity. Thus, simple cross-sectional comparisons are likely to yield a spurious positive association between onsite MRI acquisition and MRI volume owing to the endogeneity of onsite MRI capacity acquisition.

Still other past studies, such as Sharpe et al. [17], focus on imaging volume within practices acquiring imaging capacity over time, without an appropriate contemporaneous comparison group. Our results indicate that MRI volume increased over time for both MRI and non-MRI practices. Without an appropriate comparison group, our results might have suggested (incorrectly) that MRI acquisition per se was associated with an increase in MRI volume.

Finally, much of the early literature examining physician self-referral for imaging services focused on the general issue of physician investment interests in imaging facilities, including free-standing (off-site) imaging centers. As noted, organizational economics theory suggests that there are likely to be advantages (in terms of lower monitoring and transactions costs) associated with the ownership of imaging capacity for providers making more extensive use of imaging in their practices, compared to less imaging-intensive providers. However, these advantages are likely to more substantive for onsite capacity compared to off-site capacity. In other words, the degree of organizational control may be somewhat greater for owned off-site capacity compared to non-owned offsite capacity, but the degree of organizational control is likely to be far greater for owned onsite capacity compared to owned off-site capacity. Thus, the process of physician self-selection into ownership of onsite imaging capacity reflected in our data may be different than the process of self-selection into imaging capacity ownership overall present in older studies.

### Limitations

Although we used a web-based survey of orthopedic surgery practices to identify specific providers affiliated with practices at the time the practice first acquired onsite MRI capacity, and then used the CMS National Plan and Provider Enumeration System (NPPES) Full Replacement Monthly NPI File data and a re-survey of the final sample of practices included in the analysis to confirm that physicians identified as affiliated with an MRI practice in the survey data actually were affiliated with the practice one year before and one year the practice’s MRI-year, the potential for errors in assignment of specific physicians to specific practices remains. If these assignment errors are common, the results of the claims data analysis of the change in MRI volume would be biased toward a finding of “no effect” of onsite MRI capacity.

While the practice re-survey confirmed 90 % of provider practice affiliations, the re-survey response rate was 43 %, so a similar rate of confirmation might not have been obtained from practices not responding to the re-survey. However, the fact that model results restricted to a sample of providers with confirmed practice affiliations produced results similar to results using the full (principal) provider sample provides some assurance that the potential for provider assignment errors is not a substantial limitation of the study.

The sample of providers included in the study was derived from a PS matching approach applied at the practice level using a specific caliper intended to provide a reasonable trade-off between covariate balance and the number of MRI practices retained in the final sample. Selection of a smaller caliper would have produced fewer matches, and thus fewer providers in our analysis sample, whereas a larger caliper would have produced more matches, and thus a larger provider sample. It is possible that a different practice-level PS matching approach yielding a different sample of providers in MRI and non-MRI practices would have produced different results. However, the fact that model results using the full (principal) provider sample were similar to model results using a sample of providers with re-survey confirmed practice affiliations suggests that the results are not highly sensitive to sampling approach used to select the specific providers included in the analysis.

Obviously, our analysis of Medicare claims data only provides information about patterns of MRI use within the Medicare segment of each physician’s patient population. No inference about whether onsite MRI acquisition affects patterns of MRI use for other payers is possible. Past studies have shown that geographic variation in the use of specific services for Medicare patients is not always reflective of patterns of use in non-Medicare populations [48]. Orthopedic surgery practices on average derive about one-third of their total practice revenues from Medicare. While this is not an inconsequential share, this study cannot assess the impact of onsite MRI capacity on use patterns for about two-thirds of the typical orthopedic surgery practice population. Even so, an assessment of the impact of onsite MRI capacity on use patterns for Medicare patients has direct relevance for public policy, as the Stark laws only apply to Medicare and Medicaid patients.

Moreover, commercial payers, especially managed care plans, typically employ stricter MRI utilization controls and incentives than the Medicare program [49]. Thus, rather than a limitation, our choice of examining the Medicare population could alternatively be viewed as a conservative decision; if provider ownership in onsite imaging capacity has a causal impact on imaging volume, we would expect the magnitude of the effect to be larger in the comparatively “less managed” Medicare population relative to more active care management in managed care markets. Our null finding for the Medicare population suggests the likelihood of a null finding in managed care population

## Conclusion

Our analysis of Medicare claims data employed outpatient claims data for the 2007, 2008, and 2009 cohorts of physicians in practices which acquired onsite MRI capacity and physicians in matched non-MRI practice. The claims analysis focused on the change in Medicare MRI volume one-year post-onsite-MRI-acquisition and one-year pre-onsite-MRI-acquisition for physicians in MRI practices relative to physicians in the non-MRI comparison practices. In all of the Medicare MRI volume change models estimated, the estimated impact of onsite MRI acquisition on the change in Medicare MRI volume is consistently small and not statistically significant. Thus, our data analysis provides no empirical support for the proposition that acquisition of onsite MRI capacity within an orthopedic surgery practice induces an increase in the rate of MRI use for Medicare patients among practice providers, relative to physicians in practices without MRI capacity over the same time period.
